# Weight status, percent body fat, eating habits, depression, and food addiction in Palestinian university students: a cross-sectional study

**DOI:** 10.1186/s12889-026-28922-9

**Published:** 2026-08-05

**Authors:** Hazem Agha, Walaa Maher Idrees, Raghda Amro

**Affiliations:** 1 Faculty of Public Health, Al-Quds University, Jerusalem, Palestine; 2 College of Medicine and Health Sciences, Palestine Polytechnic University, Hebron, Palestine

**Keywords:** BMI, PBF, Eating habits, Food addiction, Depression, Socio-demographic, University students

## Abstract

**Background:**

Obesity and excess adiposity among university students are important public health concerns. This study aimed to determine the prevalence of overweight and obesity among Al-Quds University students using both body mass index (BMI) and percent body fat (PBF), and to examine their associations with eating habits, depressive symptoms, and food addiction.

**Methods:**

A cross-sectional study was conducted among 300 Al-Quds University students aged 18 years and older. Data were collected using a structured questionnaire that assessed sociodemographic characteristics, eating habits, depressive symptoms, and food addiction. Height and weight were measured to calculate BMI, and body composition was assessed to determine PBF. Descriptive statistics, chi-square tests, Pearson correlation, and binary logistic regression were used for data analysis.

**Results:**

Based on BMI classification, 31.7% of participants were classified as having overweight or obesity, whereas 61.0% were classified as over fat according to PBF. Male students had a significantly higher prevalence of overweight/obesity than female students according to BMI (43.2% vs. 24.2%, *P* < 0.001), while female students had a significantly higher prevalence of excess body fat than male students according to PBF (71.4% vs. 44.9%, *P* < 0.001). Eating-habits category was not significantly associated with BMI or PBF, and depressive symptoms were not significantly associated with either adiposity indicator. However, eating habits were significantly associated with depression level (*P* = 0.029). In multivariable analysis, male sex (adjusted odds ratio [aOR] = 2.28, 95% confidence interval [CI]: 1.30–3.98), older age (aOR = 1.74, 95% CI: 1.04–2.91), and severe food addiction (aOR = 8.06, 95% CI: 2.01–32.37) were associated with higher odds of overweight/obesity.

**Conclusion:**

Excess adiposity was common among Al-Quds University students, and PBF identified a substantially greater proportion of students with excess adiposity than BMI alone. Male sex, older age, and severe food addiction were associated with overweight/obesity, whereas general eating-habits category and depressive symptoms were not independently associated with adiposity in the reported analyses. These findings support the value of combining BMI with body-composition assessment in university populations and highlight the need for integrated university-based health strategies.

**Supplementary Information:**

The online version contains supplementary material available at https://doi.org/10.1186/s12889-026-28922-9.

## Introduction

Obesity is a major and growing global public health challenge. According to the World Health Organization (WHO), 2.5 billion adults aged 18 years and older were overweight in 2022, including 890 million living with obesity; overall, 43% of adults were overweight and 16% were living with obesity [1]. Obesity is strongly linked to noncommunicable diseases and remains a major contributor to morbidity and mortality worldwide [2].

University students represent an important population for obesity research because the transition to university is often accompanied by changes in autonomy, daily routines, food choices, physical activity, sleep, and stress [3]. Recent review show that weight-related outcomes in university students are shaped by multiple interacting factors, including dietary patterns, low physical activity, sedentary behavior, sleep problems, psychosocial stress, and aspects of the campus environment, rather than diet alone [4].

Within this multifactorial context, eating-related and psychological factors deserve particular attention. Food addiction has been increasingly studied as a behavioral construct that may be associated with overeating and excess weight, while depression and related mental health problems are also common in student populations. Recent review evidence from the region suggests that obesity in university students should be examined together with mental health and lifestyle-related variables, rather than as an isolated nutritional problem [5].

Body mass index (BMI) is widely used in epidemiological research because it is simple and practical, but it does not distinguish fat mass from lean mass and may therefore misclassify some individuals [6]. Percent body fat (PBF) provides complementary information on body composition and may identify excess adiposity that BMI alone does not detect. Against this background, the present study aimed to assess overweight and obesity among Al-Quds University students using both BMI and PBF, and to examine their associations with eating habits, depression, and food addiction.

## Methods

### Study design and setting

A cross-sectional study was conducted at the Nutritional Assessment Laboratory, Al-Quds University, Jerusalem, Palestine. The study aimed to determine the prevalence of overweight and obesity among university students using both body mass index (BMI) and percent body fat (PBF), and to examine their associations with eating habits, depressive symptoms, and food addiction.

### Participants and sampling

The study population comprised male and female Al-Quds University students aged 18 years and older. Participants were recruited from university faculties using a non-probability convenience sampling approach. The required sample size was estimated using Epitools (Ausvet), based on an assumed prevalence of 25%, 95% confidence level, 5% precision, and a source population of 12,712 students, yielding a minimum sample of 289 students. A total of 300 students were ultimately enrolled. The majority of participants were female (60.7%), aged 18–20 years (70.3%), single (90.7%), and enrolled in health-related faculties (87.7%). Most students resided in cities or villages, and 85.7% reported living in owner-occupied housing.

### Data collection and study measures

Data were collected using a structured self-administered questionnaire together with anthropometric and body-composition assessment. The questionnaire included four components. The first component collected socio-demographic information, including sex, parental education, household income, place of residence, and housing status. The second component assessed eating habits using a 15-item instrument evaluating the frequency of consumption of selected food items. Total scores were categorized as healthy (37–48 points), moderate (13–36 points), or unhealthy (0–12 points). The third component assessed food addiction using the modified Yale Food Addiction Scale 2.0 (mYFAS 2.0), a 13-item instrument based on DSM-5 criteria and rated on an 8-point Likert scale. The fourth component assessed depressive symptoms using the Beck Depression Inventory (BDI), which classifies symptom severity from minimal to extreme. The questionnaire was piloted prior to data collection and demonstrated acceptable internal consistency (Cronbach’s alpha = 0.80).

After questionnaire completion, anthropometric measurements were obtained using a BSM 370 stadiometer (Biospace Co., Seoul, Korea) to measure height and weight. BMI was calculated as weight in kilograms divided by height in meters squared (kg/m²). Body composition was then assessed using the InBody 120 analyzer (Biospace Co., Seoul, Korea), from which PBF was obtained. BMI and PBF were subsequently categorized according to established clinical criteria.

### Ethical considerations

The study was approved by the Ethics Committee of the School of Public Health, Al-Quds University. All participants were informed about the aims of the study, the voluntary nature of participation, and the measures taken to preserve privacy and confidentiality. Written informed consent was obtained from all participants before data collection, and participants were informed of their right to decline participation or withdraw at any time without consequence.

### Statistical analysis

Data were entered and analyzed using IBM SPSS Statistics version 25. Continuous variables were summarized as mean ± standard deviation (SD), whereas categorical variables were presented as frequencies and percentages. Between-group comparisons were performed using chi-square tests for categorical variables and independent-samples t-tests for continuous variables, as appropriate. Pearson correlation coefficients were used to assess the relationships among BMI, PBF, food addiction, depressive symptoms, and eating-habits scores. Binary logistic regression analysis was conducted to identify factors independently associated with overweight/obesity. Variables entered into the multivariable model included sex, age, eating-habits category, food addiction, and housing status, based on the univariate analysis and the study objectives. Statistical significance was set at a two-sided P value < 0.05.

## Results

A total of 300 Al-Quds University students were included in the analysis. Females comprised 60.7% of the study sample, and most participants were aged 18–20 years (70.3%). The majority were single (90.7%), lived in owned housing (85.7%), and were enrolled in health-related faculties (87.7%). Additional sociodemographic characteristics of the study population are presented in Table 1.

**Table 1:** Sociodemographic characteristics of the participants (*n* = 300)

Variable	Category	*n*	%
Gender	Male	118	39.3
	Female	182	60.7
Age (years)	18–20	211	70.3
	21–24	79	26.3
	> 24	10	3.3
Marital status	Married	22	7.3
	Single	272	90.7
	Widow	4	1.3
	Divorced	2	0.7
Place of residence	Village	137	45.7
	City	147	49.0
	Camp	16	5.3
Ownership status of house	Owned	257	85.7
	Rented	17	5.7
	Student hostels	26	8.7
Monthly income	< 1500 NIS	28	9.3
	> 1500 NIS	50	16.7
	> 3000 NIS	96	32.0
	> 5000 NIS	126	42.0
Faculty	Health related	263	87.7
	Non-health related	37	12.3
Father’s education	Less than high school	68	22.7
	High school	114	38.0
	Diploma	8	2.7
	Bachelor’s degree	68	22.7
	Master’s degree	18	6.0
	PhD	24	8.0
Mother’s education	Less than high school	60	20.0
	High school	108	36.0
	Diploma	20	6.7
	Bachelor’s degree	78	26.0
	Master’s degree	19	6.3
	PhD	15	5.0

Percentages may not total 100.0 exactly because of rounding

According to BMI classification, 62.7% of participants were in the underweight/normal-weight category, whereas 31.7% were classified as having overweight or obesity. Specifically, 23.0% were overweight and 8.6% were obese. In contrast, classification based on percent body fat (PBF) showed that 61.0% of students were categorized as over fat, while 39.0% were classified as under/normal fat. These findings indicate that PBF identified a substantially larger proportion of students with excess adiposity than BMI alone (Table 2).

**Table 2:** BMI and PBF classification by sex (*n* = 300)

Characteristic	Total (*n* = 300)	Male (*n* = 118)	Female (*n* = 182)	*P*-value
BMI category, n (%)				< *0.001*
Under/Normal weight	205 (68.3)	67 (56.8)	138 (75.8)	
Overweight/Obesity	95 (31.7)	51 (43.2)	44 (24.2)	
BMI (kg/m²), mean ± SD	23.8 ± 4.3	24.9 ± 4.4	23.1 ± 4.1	0.403
PBF category, n (%)				< *0.001*
Under/Normal fat	117 (39.0)	65 (55.1)	52 (28.6)	
Over fat	183 (61.0)	53 (44.9)	130 (71.4)	
PBF (%), mean ± SD	27.8 ± 9.5	20.4 ± 8.3	32.6 ± 6.8	0.013

*Abbreviations*: *BMI* body mass index, *PBF* percent body fat, *SD* standard deviation

Significant sex differences were observed for both adiposity indicators. Male students had a significantly higher prevalence of overweight/obesity than female students according to BMI classification (43.2% vs. 24.2%, *P* < 0.001). In contrast, female students had a significantly higher prevalence of over-fat classification than male students according to PBF (71.4% vs. 44.9%, *P* < 0.001). Mean PBF was also significantly higher among females than males (32.6 ± 6.8 vs. 20.4 ± 8.3, *P* = 0.013), whereas no statistically significant sex difference was reported in mean BMI (Table 2).

With regard to lifestyle and psychological characteristics, most participants were classified as having moderate eating habits (76.0%), while 17.0% had unhealthy eating habits and 7.0% had healthy eating habits. More than half of the students (56.7%) had no depression, 31.0% had mild-to-borderline depressive symptoms, and 12.3% had moderate-to-severe depressive symptoms. Food addiction was uncommon overall, with 91.0% of participants classified as having no food addiction (Tables 3 and 4).

**Table 3:** Bivariate associations of eating habits, depression, and food addiction with BMI and PBF categories (*n* = 300)

Variable	Category	Under/Normal Weight *n* (%)	Overweight/Obesity *n* (%)	*P*-value	Under/Normal Fat *n* (%)	Over Fat *n* (%)	*P*-value
Eating habits	Healthy	15 (5.0)	6 (2.0)	0.526	7 (2.3)	14 (4.7)	0.563
	Moderate	152 (50.7)	76 (25.3)		87 (29.0)	141 (47.0)	
	Unhealthy	38 (12.7)	13 (4.3)		23 (7.7)	28 (9.3)	
Depression	No depression	120 (40.0)	50 (16.7)	0.629	67 (22.3)	103 (34.3)	0.431
	Mild to borderline	61 (20.3)	32 (10.7)		39 (13.0)	54 (18.0)	
	Moderate to severe	24 (8.0)	13 (4.3)		11 (3.7)	26 (8.7)	
Food addiction	No FA	191 (63.7)	82 (27.3)	0.053	107 (35.7)	166 (55.3)	0.975
	Mild FA	4 (1.3)	3 (1.0)		3 (1.0)	4 (1.3)	
	Moderate FA	6 (2.0)	2 (0.7)		3 (1.0)	5 (1.7)	
	Severe FA	4 (1.3)	8 (2.7)		4 (1.3)	8 (2.7)	

*Abbreviations*: *BMI* body mass index, *PBF* percent body fat, *FA* food addiction

**Table 4:** Relationship between eating habits and depression level (*n* = 300)

Depression level	Healthy EH *n* (%)	Moderate EH *n* (%)	Unhealthy EH *n* (%)	Total *n* (%)	*P*-value
No depression	11 (3.7)	120 (40.0)	39 (13.0)	170 (56.7)	0.029
Mild to borderline	6 (2.0)	79 (26.3)	8 (2.7)	93 (31.0)	
Moderate to severe	4 (1.3)	29 (9.7)	4 (1.3)	37 (12.3)	

*Abbreviation*: *EH* eating habits

Bivariate analysis showed no statistically significant association between eating-habits category and BMI category (*P* = 0.526), nor between eating-habits category and PBF category (*P* = 0.563). Similarly, depression category was not significantly associated with BMI category (*P* = 0.629) or PBF category (*P* = 0.431). For food addiction, the association with BMI approached statistical significance (*P* = 0.053), whereas no significant association was observed with PBF category (*P* = 0.975). Nevertheless, a notable pattern was observed in the severe food-addiction group, in which 66.7% of students were classified as having overweight/obesity, compared with 30.0% of those with no food addiction (Table 3).

A statistically significant association was identified between eating habits and depression level (*P* = 0.029). Students without depression were more likely to report healthy eating habits than those with moderate-to-severe depressive symptoms. Across all depression categories, the majority of students were classified as having moderate eating habits (Table 4).

In the univariate analysis, sex was significantly associated with overweight/obesity, and age was also significantly associated with BMI category (*P* = 0.011). Housing type was associated with BMI category (*P* = 0.025), whereas eating habits did not show a significant univariate association (*P* = 0.526). Although the overall univariate association between food addiction and BMI did not reach conventional statistical significance (*P* = 0.053), severe food addiction showed a marked difference in the proportion of overweight/obesity compared with the reference group (Table 5).

**Table 5:** Univariate and multivariable analysis of factors associated with overweight/obesity

Predictor	Category	% in overweight/obesity group (univariate)	*P*-value	Adjusted OR (95% CI)	*P*-value
Gender	Male	43.2	< 0.001	2.28 (1.30–3.98)	0.004
	Female (Ref)	24.2		1.00	
Age (years)	18–20	28.0	0.011	1.74 (1.04–2.91)	0.034
	21–24	36.7			
	> 24	70.0			
Eating habits	Healthy	28.6	0.526	1.20 (0.41–3.57)	0.739
	Moderate	33.3		0.98 (0.28–3.42)	0.977
	Unhealthy (Ref)	25.5		1.00	
Food addiction	Severe	66.7	0.053	8.06 (2.01–32.37)	0.003
	None (Ref)	30.0		1.00	
Housing	Student hostels (Ref)	50.0	0.025	1.00	
	Rented	17.6		0.33 (0.13–0.86)	0.023
	Owned	30.7		0.12 (0.02–0.64)	0.013

Age is presented in categories in the univariate column, whereas a single adjusted OR is reported in the manuscript for the multivariable model. This should be clarified in the final submitted version

*Abbreviations*: *OR* odds ratio, *CI* confidence interval, *Ref* reference category

In the multivariable logistic regression model, male sex was independently associated with higher odds of overweight/obesity compared with female sex (adjusted odds ratio [aOR] = 2.28, 95% CI: 1.30–3.98, *P* = 0.004). Older age was also associated with higher odds of overweight/obesity (aOR = 1.74, 95% CI: 1.04–2.91, *P* = 0.034). Severe food addiction was the strongest independent correlate, with affected students having more than eightfold greater odds of overweight/obesity than students with no food addiction (aOR = 8.06, 95% CI: 2.01–32.37, *P* = 0.003). In contrast, eating-habits category was not independently associated with overweight/obesity. Housing status was also associated with the outcome, as students living in rented accommodation (aOR = 0.33, 95% CI: 0.13–0.86, *P* = 0.023) or owned housing (aOR = 0.12, 95% CI: 0.02–0.64, *P* = 0.013) had lower odds of overweight/obesity than students living in hostels (Table 5).

When factors associated with high PBF were examined, BMI category was reported as the only significant predictor, with students in the overweight/obesity group having 1.81 times higher odds of also being classified as over fat. This finding further supports the close, although not identical, relationship between BMI and body-fat classification (Table 5).

Pearson correlation analysis demonstrated a moderate positive correlation between BMI and PBF (*r* = 0.505, *P* < 0.001). Food addiction was positively correlated with BMI (*r* = 0.193, *P* = 0.001) and with depression (*r* = 0.150, *P* = 0.009). Eating-habits score was negatively correlated with depression (*r* = -0.192, *P* = 0.001). No significant correlations were observed between eating habits and BMI, eating habits and PBF, or food addiction and PBF (Table 6).

**Table 6:** Pearson correlation coefficients between body composition measures, eating behaviors, and psychological factors (*N* = 300)

Variable	1. BMI	2. PBF	3. Food Addiction	4. Depression	5. Eating Habits
1. BMI	—				
2. Percent Body Fat (PBF)	0.505**	—			
3. Food Addiction (FA)	0.193**	0.025	—		
4. Depression	0.014	0.044	0.150*	—	
5. Eating Habits (EH)	-0.095	-0.080	-0.078	-0.192**	—

*Abbreviations*: *BMI* body mass index, *PBF* percent body fat, *FA* food addiction, *EH* eating habits

*P < 0.05; **P < 0.01

## Discussion

This cross-sectional study examined adiposity among Al-Quds University students using BMI and assessed their associations with eating habits, depressive symptoms, and food addiction. The main findings were fourfold. First, excess adiposity was common in this sample. Second, PBF identified a substantially larger proportion of students with excess adiposity than BMI alone. Third, male sex and older age were associated with higher odds of overweight/obesity by BMI, while females showed a higher prevalence of excess adiposity by PBF. Fourth, severe food addiction was strongly associated with overweight/obesity, whereas general eating-habits category and depression were not independent correlates of adiposity in the reported multivariable model. These findings suggest that adiposity in this population is associated with a combination of body-composition, sociodemographic, and psychological factors rather than with broad dietary pattern categories alone.

One of the most important findings of this study is the discrepancy between BMI- and PBF-based classification. While 31.7% of participants were classified as having overweight or obesity by BMI, 61.0% were classified as over-fat by PBF. In addition, the moderate positive correlation between BMI and PBF indicates that the two measures are related but not interchangeable. This pattern supports the rationale for including body-composition measures alongside BMI in young adult populations, where BMI may not fully reflect adiposity. The finding is also consistent with WHO’s emphasis that obesity reflects excess fat accumulation rather than body weight alone [7]. In practical terms, the present results suggest that reliance on BMI alone may underestimate excess adiposity in some students, particularly in female students, who in this study had a markedly higher prevalence of excess body fat despite a lower prevalence of overweight/obesity by BMI.

The observed sex differences are also noteworthy. Male students had a higher prevalence of overweight/obesity by BMI, whereas female students had a substantially higher prevalence of excess adiposity by PBF. This pattern may reflect sex-related differences in body composition and fat distribution, and it highlights the importance of interpreting BMI with caution when comparing males and females. The present findings are also broadly consistent with prior Palestinian data showing higher BMI-defined overweight/obesity among male university students, although direct comparison should be made carefully because study populations, measurement approaches, and sampling strategies differ across studies [8]. Likewise, older age was associated with higher odds of overweight/obesity in the multivariable model, which may reflect cumulative behavioral and environmental exposures during the university years; however, because the study is cross-sectional, the temporal direction of this association cannot be determined.

Another important finding is that severe food addiction showed the strongest association with overweight/obesity in the multivariable analysis, whereas the broader eating-habits score did not. In addition, food addiction was positively correlated with BMI and with depressive symptoms. These results suggest that addictive-like eating behaviors may capture a dimension of eating-related risk that is not adequately represented by a general dietary-pattern score. Recent studies in university and young-adult populations have similarly reported that food addiction is associated with higher body weight and adverse psychological characteristics [9]. At the same time, this result should be interpreted cautiously. The number of students in the severe food-addiction category was small, and the confidence interval for the adjusted odds ratio was wide, which indicates statistical uncertainty around the effect estimate. Accordingly, the finding is best interpreted as a signal of a potentially important association that merits confirmation in larger studies rather than as definitive evidence of a dominant mechanism.

In contrast, neither the eating-habits category nor depression category showed a statistically significant independent association with BMI or PBF in the reported analyses. Nevertheless, eating habits were significantly associated with depression, and the correlation analysis showed that higher depressive symptoms were associated with poorer eating-habits scores and higher food-addiction scores. These findings suggest that depression may be more closely linked to eating-related behaviors than to adiposity itself in this dataset. This interpretation is consistent with recent regional review evidence indicating that the relationship between obesity and mental health in university students is multifactorial and shaped by a broader set of influences, including sleep, stress, body image, sociocultural factors, and access to support services [10]. It is therefore possible that the associations between depression and adiposity in this study were attenuated by unmeasured factors or by limitations of sample size and study design.

The association observed between housing type and overweight/obesity should also be interpreted carefully. Students living in hostels had higher odds of overweight/obesity than those living in rented or owned housing. Although this may indicate that living environment is relevant to student health, the current study did not collect detailed data on food access, meal patterns, cooking facilities, commuting, physical activity, sleep, or other contextual features that could explain this association [11]. As a result, this finding should be considered exploratory. More broadly, recent reviews indicate that obesity-related outcomes in university students are shaped by multiple modifiable determinants, including dietary habits, sleep quality, screen time, physical activity, sedentary behavior, stress, and broader environmental barriers and facilitators. This reinforces the view that the current findings should be interpreted within a broader behavioral and environmental framework rather than being attributed to any single factor [12, 13].

This study has several limitations that should be acknowledged explicitly. First, the cross-sectional design precludes causal inference and does not allow temporal ordering of adiposity, depression, food addiction, or eating habits. Second, participants were recruited using non-probability convenience sampling from a single university, and 87.7% were from health-related faculties; this introduces selection bias and limits generalizability to the broader Palestinian university population. Third, several exposures were self-reported, which raises the possibility of recall bias and social-desirability bias. Fourth, some relevant determinants of adiposity were not measured, including physical activity, sedentary behavior, sleep, stress, smoking, alcohol use, medication use, and detailed characteristics of the campus and housing environment. Fifth, some analytical subgroups were small, particularly the severe food-addiction category, which may have reduced precision and produced unstable regression estimates. Sixth, although PBF strengthens the assessment of adiposity, body-composition classification may still be affected by the specific cut-offs and device-based measurement approach used.

Despite these limitations, the study has important strengths. It addresses an under-researched Palestinian university population, uses direct anthropometric and body-composition assessment rather than BMI alone, and evaluates psychological and behavioral variables together. Overall, the findings indicate that excess adiposity is common among Al-Quds University students, that PBF may identify additional at-risk students beyond BMI, and that severe food addiction appears to be an important correlate of BMI-defined overweight/obesity in this sample. These results support the value of university-based health strategies that combine nutrition education with broader student-health approaches, including mental-health support, behavioral screening, and attention to the campus living environment. Future studies should use larger and more representative samples, collect data on physical activity, sleep, sedentary behavior, and environmental exposures, and apply longitudinal designs to better clarify temporal relationships among adiposity, eating behaviors, and psychological factors.

## Conclusion

Excess adiposity was common among Al-Quds University students, and percent body fat identified more students with excess adiposity than body mass index alone. Male sex, older age, and severe food addiction were associated with overweight/obesity, while eating-habits category and depressive symptoms were not independently associated with adiposity in the adjusted analysis. These findings support the value of combining BMI with body-composition assessment in university populations. Given the cross-sectional design and sampling limitations, further longitudinal studies with more representative samples are warranted.

## Supplementary Information

Supplementary Material 1.

## Data Availability

Additional Supplementary Table 1. Data are available from the corresponding author upon reasonable request.

## Abbreviations

BMI: Body Mass Index
PBF: Percent Body Fat
WHO: World Health Organization
FA: Food Addiction
BF: Body Fat
NIS: New Israeli Shekel
DSM-5: Diagnostic and Statistical Manual of Mental Disorders, Fifth Edition
mYFAS 2.0: Modified Yale Food Addiction Scale 2.0
BDI: Beck Depression Inventory
BSM 370: Portable Automatic Stadiometer
SPSS: Statistical Package for Social Sciences
SD: Standard Deviation
EH: Eating Habits
aOR: Adjusted Odd Ratio
CI: Confidence intervals
R: PEARSON Correlation
