# Supplementary material for: Weight status, percent body fat, eating habits, depression, and food addiction in Palestinian university students: a cross-sectional study
Source: BMC Public Health. 2026 Aug 5;26:3157. doi: 10.1186/s12889-026-28922-9 (PMC13726003; doi:10.1186/s12889-026-28922-9)
Supplement: Supplementary file 1 [file 12889_2026_28922_MOESM1_ESM.docx]

**Research Questionnaire**

**A1. Questionnaire Serial Number (Code): _______________.**

**Section One: Personal Information**

A2 Name: _____________________ (*Name will not appear in the research).*

A3 Personal Phone Number: _________________

**Section Two: Anthropometric Measurements**
*This section will be completed by the researcher:*

| Weight (kg)………………….. | B1 |
| --- | --- |
| Height (cm)………………….. | B5 |
| BMI (Body Mass Index)……………….. | B6 |
| PBF (Percent Body Fat)……………………….. | B7 |

**Section Three: Basic Information**

| Code | Question | Options |
| --- | --- | --- |
| C1 | Faculty: | ............................................ |
| C2 | Specialization: | 1. Literary (Arts) 2. Scientific |
| C3 | Gender: | 1. Male 2. Female |
| C4 | Age: | 18-20 21-24 >24 |
| C5 | Marital Status: | 1. Married 2. Single 3. Widowed 4. Divorced |
| C6 | If you are married, are you? | 1. Pregnant 2. Breastfeeding 3. No |
| C7 | Current family residence: | 1. Village 2. City 3. Refugee Camp |
| C8 | Type of housing: | 1. Owned 2. Rented 3. University Dormitory |
| C9 | Family income (in Shekels): | 1. Less than 1500 2. More than 1500 3. More than 3000 4. More than 5000 |
| C10 | Father's years of education (including school): | ............................ |
| C11 | Mother's years of education (including school): | ............................ |

**Section Four: Dietary Habits Questionnaire**

|  | Question | Always | Most of the time | Rarely | Never |
| --- | --- | --- | --- | --- | --- |
| D1 | Do you eat vegetables or salad with one or more meals per day? |  |  |  |  |
| D2 | Do you eat at least two servings of fruit daily? |  |  |  |  |
| D3 | Do you try to avoid refined carbohydrates (starches) like white bread, white rice, sweetened breakfast cereals? |  |  |  |  |
| D4 | Do you try to remove fat from meat and skin from chicken before cooking? |  |  |  |  |
| D5 | Do you use cooking methods to reduce fat quantity, such as grilling, boiling, dry roasting, or microwaving? |  |  |  |  |
| D6 | Do you eat fish or seafood (fresh or canned) once or twice a week? |  |  |  |  |
| D7 | Do you add salt to your food at the table? |  |  |  |  |
| D8 | Do you buy salt-free or low-salt foods like cheeses, biscuits, sauces, or others? |  |  |  |  |
| D9 | Do you try to limit sweetened carbonated drinks or concentrated fruit juices? |  |  |  |  |
| D10 | Do you prefer to eat chocolate, cake, sweets, only on special occasions? |  |  |  |  |
| D11 | Do you maintain moderate caffeine intake (less than 2 cups of coffee, or 2 cups of energy drink, or 4 cups of tea per day)? |  |  |  |  |
| D12 | Do you choose low-fat fast food options (e.g., burger, shawarma wraps, sandwiches with little or no fat) and eat plenty of salad with them? |  |  |  |  |
| D13 | Do you eat breakfast in the morning? |  |  |  |  |
| D14 | Do you eat when you are hungry and stop when you are comfortably full? |  |  |  |  |
| D15 | Do you eat a variety of different foods throughout the day? |  |  |  |  |

**Section Five: The Yale Food Addiction Scale (YFAS)**
This questionnaire asks about your eating habits over the past year. People sometimes find it difficult to control their intake of certain foods, such as:

- **Sweets:** Ice cream, chocolate, doughnuts, cookies, cake, candy.
- **Starches:** White bread, pastries, pasta, rice.
- **Salty snacks:** Potato chips, corn chips (e.g., Doritos), crackers, salty biscuits.
- **Fatty foods:** Steak, hamburger, cheeseburger, pizza, French fries.
- **Sugary drinks:** Sodas, sports drinks, energy drinks.

When the following items refer to "certain foods," please think of any foods or drinks similar to those in the groups listed above, or any other foods with which you have had difficulty in the past year.

|  | During the past 12 months... | Never | Less than monthly | Once a month | 2-3 times a month | Once a week | 2-3 times a week | 4-6 times a week | Daily |
| --- | --- | --- | --- | --- | --- | --- | --- | --- | --- |
| E1 | I ate to the point of feeling physically ill. |  |  |  |  |  |  |  |  |
| E2 | I spent a lot of time feeling sluggish or tired from overeating. |  |  |  |  |  |  |  |  |
| E3 | I avoided work, school, or social activities because I was afraid I would overeat there. |  |  |  |  |  |  |  |  |
| E4 | When I felt moody (e.g., sad, stressed, nervous, irritable) because I hadn't eaten certain foods, I ended up eating them to feel better. |  |  |  |  |  |  |  |  |
| E5 | My eating style caused me significant psychological distress (e.g., feeling upset, stressed, self-loathing, guilt). |  |  |  |  |  |  |  |  |
| E6 | I had significant problems in my life due to food and my eating style; these could be problems with daily routine, work/school, friends, family, or health troubles. |  |  |  |  |  |  |  |  |
| E7 | My overeating interfered with my ability to care for my family or perform household tasks. |  |  |  |  |  |  |  |  |
| E8 | I continued to eat in the same way even though it was causing me emotional problems. |  |  |  |  |  |  |  |  |
| E9 | Eating the same amount of food didn't give me the same level of pleasure as before. |  |  |  |  |  |  |  |  |
| E10 | I had such strong urges to eat certain foods that I couldn't think of anything else. |  |  |  |  |  |  |  |  |
| E11 | I tried and failed to cut down on or stop eating certain foods. |  |  |  |  |  |  |  |  |
| E12 | I was so distracted by eating in situations requiring attention that it could have put me in danger (e.g., while driving, crossing the street, operating machinery). |  |  |  |  |  |  |  |  |
| E13 | My friends/family members were worried and expressed concerns about my overeating. |  |  |  |  |  |  |  |  |

**Section Six: Beck's Depression Inventory (BDI)**
This form contains groups of statements. Please read each group carefully, then choose the single statement that best describes how you have been feeling **over the past week, including today**. Circle the number of the statement you choose. If several statements in a group seem to apply equally well, you may circle multiple numbers. Be sure to read all statements in each group before making your choice.

**(F1)**

1. I do not feel sad.
2. I feel sad.
3. I am sad all the time and I can’t snap out of it.
4. I am so sad or unhappy that I can’t bear it.

**(F2)**

1. I am not discouraged about the future.
2. I feel discouraged about the future.
3. I feel I have nothing to look forward to.
4. I feel the future is hopeless and that things cannot improve.

**(F3)**

1. I do not feel like a failure.
2. I feel I have failed more than I should have.
3. When I look back on my life, all I can see is a lot of failures.
4. I feel I am a complete failure as a person.

**(F4)**

1. I get as much satisfaction out of things as I used to.
2. I don’t enjoy things the way I used to.
3. I don't get real satisfaction out of anything anymore.
4. I am dissatisfied or bored with everything.

**(F5)**

1. I don’t feel particularly guilty.
2. I feel guilty a good part of the time.
3. I feel guilty most of the time.
4. I feel guilty all the time.

**(F6)**

1. I don’t feel I am being punished.
2. I feel I may be punished.
3. I expect to be punished.
4. I feel I am being punished now.

**(F7)**

1. I don’t feel disappointed in myself.
2. I am disappointed in myself.
3. I am disgusted with myself.
4. I hate myself.

**(F8)**

1. I don’t feel worse than others.
2. I criticize myself for my weaknesses or mistakes.
3. I blame myself all the time for my faults.
4. I blame myself for everything bad that happens.

**(F9)**

1. I don’t have thoughts of killing myself.
2. I have thoughts of killing myself, but I would not carry them out.
3. I would like to kill myself.
4. I would kill myself if I had the chance.

**(F10)**

1. I don’t cry more than usual.
2. I cry more than I used to.
3. I cry all the time now.
4. I used to be able to cry, but now I can’t even though I want to.

**(F11)**

1. I am not more irritated now than I ever was.
2. I am more irritated now than before.
3. I feel irritated or annoyed a good part of the time.
4. I feel irritated all the time now.

**(F12)**

1. I have not lost interest in other people.
2. I am less interested in other people than I used to be.
3. I have lost most of my interest in other people.
4. I have lost all interest in other people.

**(F13)**

1. I make decisions about as well as ever.
2. I put off making decisions more than before.
3. I have greater difficulty in making decisions than before.
4. I can’t make any decisions at all anymore.

**(F14)**

1. I don’t feel I look worse than I used to.
2. I am worried that I am looking old or unattractive.
3. I feel permanent changes in my appearance make me unattractive.
4. I believe I look ugly.

**(F15)**

1. I can work about as well as before.
2. It takes extra effort to get started doing anything.
3. I have to push myself very hard to do anything.
4. I can’t do any work at all.

**(F16)**

1. I can sleep as well as usual.
2. I don’t sleep as well as I used to.
3. I wake up one or two hours earlier than usual and find it hard to get back to sleep.
4. I wake up several hours earlier than usual and cannot get back to sleep.

**(F17)**

1. I don’t get more tired than usual.
2. I get tired more easily than before.
3. I get tired from doing almost anything.
4. I am too tired to do anything.

**(F18)**

1. My appetite is no worse than usual.
2. My appetite is not as good as it used to be.
3. My appetite is much worse now.
4. I have no appetite at all.

**(F19)**

1. I haven’t lost any weight recently.
2. I have lost more than 2 kg.
3. I have lost more than 4.5 kg.
4. I have lost more than 6.5 kg.

**(F20)**

1. I am no more worried about my health than usual.
2. I am worried about physical problems like aches, stomach pains, or headaches.
3. I am very worried about physical problems, and it is hard to think about anything else.
4. I am so worried about my physical problems that I can hardly think of anything else.

**(F21)**

1. I have not noticed any recent change in my sexual desire.
2. I have less desire for sex than before.
3. I have very little desire for sex now.
4. I have completely lost interest in sex.
